# Independent association of the CHG index with cardiometabolic multimorbidity: real-world evidence from community and clinical datasets

**DOI:** 10.3389/fpubh.2026.1967131

**Published:** 2026-10-07

**Authors:** Zexu Cui, Tianhao Lan, Min Lu, Nan Zhang, Donglin Yang, Xahzat Xuhrat, Minghong Kang, Jinglun Kuang, Dilnur Turghun, Tiancai Wen, Ping Fan, Yun Zhao

**Affiliations:** 1 School of Public Health, Shaanxi University of Chinese Medicine, Xianyang, China; 2 The First School of Clinical Medicine, Xinjiang Medical University, Urumqi, China; 3 Data Center of Traditional Chinese Medicine, China Academy of Chinese Medical Sciences, Beijing, China; 4 Department of Heart Function, First Affiliated Hospital of Xinjiang Medical University, Urumqi, China

**Keywords:** cardiometabolic multimorbidity, cholesterol–high-density lipoprotein–glucose index, aging, public health, population health management, CHARLS

## Abstract

**Background:**

Cardiometabolic multimorbidity (CMM) is an increasing public health challenge in aging populations. The cholesterol–high-density lipoprotein–glucose (CHG) index is an emerging metabolic marker, but its association with prevalent CMM has not been sufficiently evaluated across community and hospitalized populations. This study examined the association between CHG and CMM using clinical and China Health and Retirement Longitudinal Study (CHARLS) data.

**Methods:**

This cross-sectional study included 5,544 hospitalized patients from the First Affiliated Hospital of Xinjiang Medical University and 11,221 community-dwelling participants from CHARLS. CMM was defined as the presence of at least two of heart disease, stroke, and diabetes mellitus. Multivariable logistic regression was used to assess associations between CHG and CMM after adjustment for demographic, lifestyle, and clinical factors. Restricted cubic spline analyses assessed nonlinearity, while receiver operating characteristic curves evaluated discriminative performance. Subgroup and sensitivity analyses were also performed.

**Results:**

Each SD increase in CHG was associated with higher odds of prevalent CMM in both the clinical dataset (OR = 1.96, 95% CI: 1.74–2.21) and CHARLS (OR = 4.14, 95% CI: 3.46–4.94). Significant J-shaped nonlinear associations were observed in both datasets. Adding CHG to the basic model improved discrimination, with AUCs of 0.690 and 0.834 in the clinical and CHARLS datasets, respectively. A nominal smoking interaction in the clinical dataset (*p* for interaction = 0.043) was not significant after false discovery rate correction. Sensitivity analyses were generally consistent with the primary findings. Phenotype-specific analyses were broadly consistent across two-disease combinations in CHARLS, whereas the heart disease–stroke phenotype without diabetes showed a distinct pattern in the clinical dataset.

**Conclusion:**

Higher CHG was independently associated with prevalent CMM in hospitalized and community-dwelling middle-aged and older Chinese adults. Nonlinear associations were observed in both datasets, and no subgroup interaction remained significant after multiple-testing correction. CHG may provide a simple, accessible indicator of cardiometabolic burden, with potential value for risk stratification and population-level identification of individuals with greater cardiometabolic disease burden. Derived from routinely available laboratory measures, CHG may complement established cardiometabolic assessment and support population health and integrated chronic disease management in aging populations.

## 1 Introduction

Global population aging has accelerated the emergence of multimorbidity, posing a substantial threat to global health systems (1). Cardiometabolic multimorbidity (CMM) represents a prevalent and clinically important form of multimorbidity (2). CMM is specifically defined as the concurrent presence of at least two cardiometabolic diseases (CMDs), typically encompassing heart disease, diabetes mellitus, and stroke (3). Epidemiological surveys indicate that the prevalence of CMM among older adults in China has increased from 5.94 to 16.9% and continues to increase rapidly (4, 5). Previous studies have reported that individuals with CMM experience a greater health burden than those with a single CMD or none, including elevated all-cause mortality, accelerated cognitive decline, and reduced quality of life (6–9). Given its rising prevalence and serious health impacts, early detection and targeted interventions are essential for mitigating adverse clinical outcomes (10).

Insulin resistance (IR), characterized by reduced tissue sensitivity to the biological effects of insulin, is associated with impaired glucose uptake, hyperglycemia, and adverse cardiovascular and metabolic profiles (11–13). Identifying and quantifying IR is therefore relevant to cardiometabolic risk assessment. Although the hyperinsulinemic-euglycemic clamp (HIEG) remains the reference method for measuring IR (14), its widespread clinical application is hampered by its invasiveness, technical complexity, and high cost. Consequently, surrogate indicators such as the triglyceride–glucose (TyG) index are widely used. Derived from fasting triglycerides and blood glucose, the TyG index is used as a surrogate measure of IR (15). Previous studies have demonstrated that the TyG index is associated with the risk of various cardiometabolic diseases (16–19). Nevertheless, the TyG index reflects only limited aspects of glucolipid metabolism. Adverse outcomes of CMM, such as heart disease and stroke, depend heavily on vascular atherosclerotic progression and defective reverse cholesterol transport (20). Thus, relying solely on triglycerides may not fully capture atherogenic lipid burden.

The cholesterol–high-density lipoprotein–glucose (CHG) index, composed of total cholesterol (TC), fasting blood glucose (FBG), and high-density lipoprotein cholesterol (HDL-C) (21), has been studied as a composite marker of IR and metabolic burden. This index was initially evaluated for identifying type 2 diabetes mellitus (T2DM) and showed greater discrimination than the TyG index (21). Furthermore, the CHG index shows a positive association with cardiovascular disease (CVD) risk (22). However, previous studies investigating CHG and CMM have predominantly focused on community cohorts (23). Because community residents and hospitalized patients differ systematically in terms of comorbidity burden, baseline metabolic state, and pharmacological treatments, its association with CMM in heterogeneous clinical inpatient populations remains uncertain.

Therefore, this study aimed to systematically analyze the association between the CHG index and CMM risk among middle-aged and older adults in China using inpatient records from the First Affiliated Hospital of Xinjiang Medical University and baseline data from the China Health and Retirement Longitudinal Study (CHARLS). Using parallel analyses in the two populations, we evaluated the discriminative capacity and effect modifiers of CHG across health settings to characterize the association across healthcare settings.

## 2 Materials and methods

### 2.1 Study design and populations

This study combined two independent data sources: clinical inpatient records and the CHARLS database. Clinical data were retrospectively gathered from hospitalized patients admitted to the First Affiliated Hospital of Xinjiang Medical University between January 2019 and December 2022. Patients were excluded if they met any of the following criteria: (1) age < 45 years (*n* = 490); (2) missing values for TC, FBG, or HDL-C (*n* = 870); or (3) missing baseline CMM data (*n* = 19). Ultimately, 5,544 clinical patients were included. Ethical approval for clinical data collection was granted by the Ethics Committee of the First Affiliated Hospital of Xinjiang Medical University [K202507-28].

The CHARLS database is a nationally representative prospective longitudinal survey targeting Chinese adults aged 45 years and older (24). Publicly accessible data can be obtained at http://CHARLS.pku.edu.cn/en. From 17,705 individuals who provided blood samples at the 2011 baseline survey, participants were excluded based on: (1) age < 45 years (*n* = 494); (2) missing values for baseline TC, FBG, or HDL-C (*n* = 5,700); or (3) missing CMM diagnostic data at baseline (*n* = 290). A total of 11,221 community participants were included. The CHARLS study protocol was approved by the Biomedical Ethics Review Committee of Peking University, and all participants provided written informed consent. The participant selection flowcharts for both datasets are shown in Figure 1.

**Figure 1:**
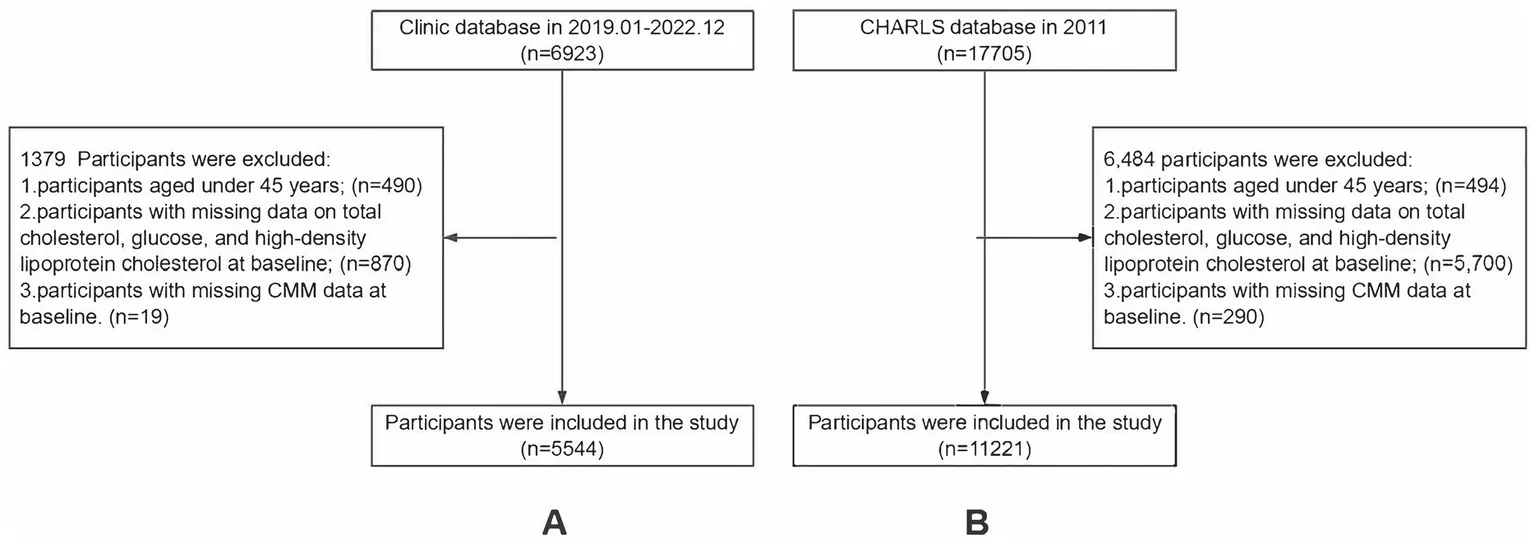
Participant selection flowcharts. **(A)** Clinical dataset. **(B)** CHARLS dataset.

### 2.2 Assessment of the CHG index

Laboratory parameters in the clinical dataset were determined using fasting blood samples collected on admission or the following morning. In CHARLS, venous blood samples were drawn after an overnight fast of at least 8 h and analyzed according to the survey protocol. Because laboratory platforms and quality-control procedures were not harmonized across datasets, absolute CHG values were not directly compared between populations. The CHG index was calculated as:

$$\mathrm{CHG}=\ln(\frac{\mathrm{TC}\;(\mathrm{mg}/\mathrm{dL})\times \mathrm{FBG}\;(\mathrm{mg}/\mathrm{dL})}{2\times \mathrm{HDL}-C\;(\mathrm{mg}/\mathrm{dL})})$$

where FBG, HDL-C, and TC represent fasting blood plasma glucose (mg/dL), high-density lipoprotein cholesterol (mg/dL), and total cholesterol (mg/dL), respectively (21).

### 2.3 Assessment of CMM

The primary study outcome was the presence of CMM, defined as the concurrent occurrence of at least two CMDs (heart disease, stroke, and diabetes) (3). In clinical data, heart disease was derived from discharge diagnoses, including coronary heart disease, heart failure, myocardial infarction, or other heart conditions. Diabetes mellitus was diagnosed based on discharge records of type 1 or type 2 diabetes. Stroke was identified through cerebrovascular accident diagnoses (such as cerebral infarction or intracerebral hemorrhage). In the CHARLS database, heart disease and stroke were identified via baseline self-reports or documented use of cardiovascular medications. Information was acquired using standardized questionnaires: “Have you ever been diagnosed by a doctor with coronary heart disease, angina, congestive heart failure, or other heart problems?” and “Have you ever been diagnosed by a doctor with a stroke?” Respondents answering “yes” were classified as having heart disease or stroke. Diabetes was confirmed if participants met any of the following criteria: (1) self-reported physician diagnosis; (2) current antidiabetic medication use; (3) FBG ≥ 126 mg/dL; or (4) glycated hemoglobin (HbA1c) ≥ 6.5% (25).

### 2.4 Covariates and data preprocessing

To control for confounding, covariates were categorized into sociodemographic factors (age, gender, educational level), lifestyle factors (smoking, alcohol drinking), medical history (stroke, heart disease, diabetes, hypertension, dyslipidemia, cancer), clinical metrics [body mass index (BMI), systolic blood pressure (SBP)], and laboratory indicators [white blood cell count (WBC), triglycerides (TG), low-density lipoprotein cholesterol (LDL-C), creatinine (CR), and uric acid (UA)].

BMI was computed as weight in kilograms divided by height in meters squared (kg/m^2^). Educational level was categorized as “less than high school” or “high school and above”. Smoking and drinking statuses were analyzed as binary variables (“Yes” or “No”). Hypertension was defined as self-reported medical history, SBP ≥ 140 mmHg, diastolic blood pressure (DBP) ≥ 90 mmHg, or current use of antihypertensive agents (26).

Missing data proportions are detailed in Supplementary Table 1. To minimize missingness bias, multivariate imputation by chained equations (MICE) with five imputed datasets was performed (27). Multicollinearity was evaluated for all variables before regression analyses (28). Significant collinearity was defined as GVIF^(1/2DF) ≥ 2, where GVIF represents the generalized variance inflation factor and DF represents degrees of freedom (Supplementary Table 2).

### 2.5 Statistical analysis

The Kolmogorov–Smirnov test was used to assess the normality of continuous distributions (29) (Supplementary Table 3). Normally distributed continuous variables are presented as mean (standard deviation, SD), and non-normally distributed continuous variables are presented as median (interquartile range, IQR). Group differences were examined using independent-samples *t* tests or Kruskal–Wallis *H* tests as appropriate. Categorical variables are reported as counts (percentages) and evaluated using Pearson’s chi-square test or the Cochran–Armitage trend test. To evaluate the potential for selection bias due to missing laboratory data, participants included in the analysis were compared with those excluded because of missing laboratory measurements.

Multivariable logistic regression models were constructed to evaluate the association between the CHG index and CMM. The CHG index was modeled both continuously (per 1-SD increment) and categorically by quartiles (Q1–Q4), with Q1 as the reference. Adjustments were implemented sequentially: Model 1 was unadjusted; Model 2 adjusted for age, gender, education level, smoking status, and drinking status; Model 3 additionally adjusted for BMI, SBP, WBC, CR, UA, hypertension, dyslipidemia, and cancer.

Restricted cubic spline (RCS) models with four knots placed at the 5th, 35th, 65th, and 95th percentiles of the CHG distribution were used to assess potential nonlinear associations between CHG and prevalent CMM. The stability of the estimated inflection points was further evaluated using 1,000 bootstrap resamples, with the inflection point re-estimated in each resample; the bootstrap median and 2.5th–97.5th percentile interval were reported. ROC curves were plotted, and area under the curve (AUC) values were calculated to compare the discriminative power of CHG, TC, FBG, and HDL-C, as well as alternative indices including TyG, METS-IR, AIP, NHHR, and RC.

Stratified analyses were conducted by age, gender, education, smoking, drinking, hypertension, dyslipidemia, and cancer, with interaction *p* values adjusted using the Benjamini–Hochberg false discovery rate procedure. Sensitivity analyses included: (1) complete-case analysis to assess the influence of missing data; (2) exclusion of individuals with baseline hypertension, dyslipidemia, or cancer to reduce potential disease-related confounding; (3) E-value analysis to evaluate the sensitivity of the associations to unmeasured confounding; (4) additional adjustment for medication use to minimize potential treatment-related confounding; and (5) three phenotype-specific analyses to assess the consistency of the associations across different CMM phenotypes.

Statistical analyses were performed using R software (version 4.5.1, R Foundation for Statistical Computing, Vienna, Austria). Two-sided *p* < 0.05 was considered statistically significant.

## 3 Results

### 3.1 Baseline characteristics

The clinical dataset comprised 5,544 patients, whose baseline features stratified by CMM status are presented in Table 1. The median age of all participants was 62.0 years (IQR: 55.0–70.0), with 3,194 (57.6%) being male. Compared to non-CMM patients, those with CMM were significantly older, included a higher proportion of men, and had a higher prevalence of alcohol use (*p* < 0.05). Participants with CMM also had higher BMI, SBP, FBG, WBC, CR, and CHG, accompanied by lower TC, HDL-C, and LDL-C levels (all *p* < 0.05). The prevalence of hypertension, diabetes, heart disease, and stroke were significantly higher in the CMM group (*p* < 0.05). Cancer was less frequent in the CMM group than in the non-CMM group (18.8% vs. 22.6%, *p* = 0.002). No significant differences were observed regarding smoking, education, dyslipidemia prevalence, TG, or UA levels.

**Table 1:** Baseline characteristics of the clinical dataset.

Characteristic	Overall	Non-CMM	CMM	*p*-value
	*N* = 5,544	*N* = 3,830	*N* = 1714	
Age, Median (IQR), Years	62.0 (55.0, 70.0)	61.0 (55.0, 69.0)	65.0 (57.0, 72.0)	<0.001
Gender, *n* (%)				0.005
Female	2,350 (42.4%)	1,672 (43.7%)	678 (39.6%)	
Male	3,194 (57.6%)	2,158 (56.3%)	1,036 (60.4%)	
Education level, *n* (%)				0.237
High school and above	2,917 (52.6%)	2,036 (53.2%)	881 (51.4%)	
Less than high school	2,627 (47.4%)	1,794 (46.8%)	833 (48.6%)	
Smoking status, *n* (%)	1,560 (28.1%)	1,051 (27.4%)	509 (29.7%)	0.090
Drinking status, *n* (%)	1,548 (27.9%)	1,038 (27.1%)	510 (29.8%)	0.045
BMI, Median (IQR), kg/m^2^	25.9 (23.5, 28.4)	25.8 (23.3, 28.3)	26.2 (23.9, 28.7)	<0.001
SBP, Median (IQR), mmHg	127.0 (119.0, 140.0)	126.0 (118.0, 140.0)	130.0 (120.0, 145.0)	<0.001
WBC, Median (IQR), ×10^9^/L	6.2 (5.2, 7.4)	6.1 (5.1, 7.3)	6.4 (5.3, 7.6)	<0.001
FBG, Median (IQR), mg/dL	95.8(84.4,119.0)	92.5(83.0,107.5)	109.8(90.0,145.4)	<0.001
TG, Median (IQR), mg/dL	127.5 (91.7, 181.6)	126.7 (91.2, 181.6)	128.9 (93.0, 182.5)	0.270
TC, Median (IQR), mg/dL	155.1 (128.8, 182.5)	158.2 (132.3, 185.6)	146.9 (120.7, 174.0)	<0.001
HDL-C, Median (IQR), mg/dL	39.4 (32.9, 47.2)	39.8 (33.3, 48.0)	38.3 (31.7, 44.9)	<0.001
LDL-C, Median (IQR), mg/dL	99.8 (76.6, 123.4)	102.9 (79.7, 125.7)	93.2 (70.4, 116.0)	<0.001
CR, Median (IQR), mg/dL	0.8 (0.7, 0.9)	0.8 (0.7, 0.9)	0.8 (0.7, 1.0)	<0.001
UA, Median (IQR), mg/dL	5.3 (4.4, 6.3)	5.3 (4.4, 6.3)	5.3 (4.5, 6.4)	0.705
Hypertension, *n* (%)	3,790 (68.4%)	2,417 (63.1%)	1,373 (80.1%)	<0.001
Dyslipidemia, *n* (%)	302 (5.4%)	210 (5.5%)	92 (5.4%)	0.912
Cancer, *n* (%)	1,188 (21.4%)	866 (22.6%)	322 (18.8%)	0.002
Diabetes, *n* (%)	2,083 (37.6%)	826 (21.6%)	1,257 (73.3%)	<0.001
Heart disease, *n* (%)	2,840 (51.2%)	1,361 (35.5%)	1,479 (86.3%)	<0.001
Stroke, *n* (%)	1,292 (23.3%)	293 (7.7%)	999 (58.3%)	<0.001
Antidiabetic medication, *n* (%)	715 (12.9%)	299 (7.8%)	416 (24.3%)	<0.001
Antidyslipidemic medication, *n* (%)	353 (6.4%)	221 (5.8%)	132 (7.7%)	0.008
CHG, Median (IQR)	6.6 (6.3, 7.0)	6.6 (6.3, 6.9)	6.7 (6.4, 7.1)	<0.001

CMM, cardiometabolic multimorbidity; BMI, body mass index; SBP, systolic blood pressure; WBC, White blood cell; FBG, fasting blood glucose; TG, triglyceride; TC, total cholesterol; HDL-C, high density lipoprotein cholesterol; LDL-C, low density lipoprotein cholesterol; CR, Creatinine; UA, uric acid.

In the CHARLS data, 11,221 participants were analyzed (Table 2). The median age of all participants was 58.0 years (IQR: 52.0–65.0), and 5,305 (47.3%) were male. Compared to the non-CMM group, CMM participants were older and exhibited higher SBP, WBC, FBG, TG, TC, UA, and CHG levels, alongside lower HDL-C levels (all *p* < 0.05). The prevalence of hypertension, dyslipidemia, diabetes, heart disease, and stroke were significantly higher in the CMM group (*p* < 0.05). Gender, education, smoking, drinking, BMI, LDL-C, and cancer prevalence did not differ significantly between groups. As shown in Supplementary Figure 1, higher CHG values were observed among participants with CMM in both datasets.

**Table 2:** Baseline characteristics of CHARLS participants.

Characteristic	Overall	Non-CMM	CMM	*p*-value
	*N* = 11,221	*N* = 10,787	*N* = 434	
Age, Median (IQR), years	58.0 (52.0, 65.0)	58.0 (51.0, 65.0)	62.0 (56.0, 69.0)	<0.001
Gender, *n* (%)				0.180
Female	5,916 (52.7%)	5,673 (52.6%)	243 (56.0%)	
Male	5,305 (47.3%)	5,114 (47.4%)	191 (44.0%)	
Education level, *n* (%)				0.555
High school and above	1,209 (10.8%)	1,158 (10.7%)	51 (11.8%)	
Less than high school	10,012 (89.2%)	9,629 (89.3%)	383 (88.2%)	
Smoking status, *n* (%)	4,455 (39.7%)	4,286 (39.7%)	169 (38.9%)	0.779
Drinking status, *n* (%)	4,657(41.5%)	4,490 (41.6%)	167 (38.5%)	0.210
BMI, Median (IQR), kg/m^2^	23.2 (20.8, 25.9)	23.1 (20.8, 25.8)	25.5 (22.5, 28.0)	0.201
SBP, Median (IQR), mmHg	127.3 (114.7, 142.0)	127.0 (114.3, 141.7)	137.0(122.3,153.0)	<0.001
WBC, Median (IQR), ×10^9^/L	6.0 (5.0, 7.2)	5.9 (4.9, 7.2)	6.2 (5.1, 7.6)	0.004
FBG, Median (IQR), mg/dL	102.6 (94.5, 113.9)	102.1(94.3,112.5)	137.2 (112.7, 167.0)	<0.001
TG, Median (IQR), mg/dL	106.2 (75.2, 156.6)	105.3 (75.2, 154.0)	144.7 (100.9, 232.8)	<0.001
TC, Median (IQR), mg/dL	190.2 (166.6, 215.7)	190.2 (166.6, 215.3)	196.6 (168.2, 223.5)	0.006
HDL-C, Median (IQR), mg/dL	49.1 (40.2, 59.5)	49.5 (40.2, 59.9)	41.8 (34.0, 50.6)	<0.001
LDL-C, Median (IQR), mg/dL	114.0 (92.8, 137.2)	114.0 (93.2, 137.2)	114.8 (90.1, 139.2)	0.368
CR, Median (IQR), mg/dL	0.8 (0.7, 0.9)	0.8 (0.7, 0.9)	0.8 (0.7, 0.9)	<0.001
UA, Median (IQR), mg/dL	4.3 (3.6, 5.2)	4.3 (3.6, 5.2)	4.6 (3.8, 5.5)	<0.001
Hypertension, *n* (%)	4,491 (40.0%)	4,177 (38.7%)	314 (72.4%)	<0.001
Dyslipidemia, *n* (%)	1,098 (9.8%)	943 (8.7%)	155 (35.7%)	<0.001
Cancer, *n* (%)	116 (1.0%)	110 (1.0%)	6 (1.4%)	0.461
Diabetes, *n* (%)	1,958 (17.4%)	1,566 (14.5%)	392 (90.3%)	<0.001
Heart disease, *n* (%)	1,403 (12.5%)	1,024(9.5%)	379 (87.3%)	<0.001
Stroke, *n* (%)	267 (2.4%)	147(1.4%)	120 (27.6%)	<0.001
Antidiabetic medication, *n* (%)	464 (4.1%)	313 (2.9%)	151 (34.8%)	<0.001
Antidyslipidemic medication, *n* (%)	623 (5.6%)	513 (4.8%)	110 (25.3%)	<0.001
CHG, Median (IQR)	5.3 (5.0, 5.6)	5.3 (5.0, 5.6)	5.8 (5.4, 6.2)	<0.001

CMM, cardiometabolic multimorbidity; BMI, body mass index; SBP, systolic blood pressure; WBC, White blood cell; FBG, fasting blood glucose; TG, triglyceride; TC, total cholesterol; HDL-C, high density lipoprotein cholesterol; LDL-C, low density lipoprotein cholesterol; CR, Creatinine; UA, uric acid.

Participants included in the analyses were generally similar to those excluded because of missing CHG components, although some differences were observed. In the clinical dataset, the largest imbalance was observed for diabetes (SMD = 0.227), whereas in CHARLS, greater differences were observed for diabetes (SMD = 0.376), creatinine (SMD = 0.345), and LDL-C (SMD = 0.308) (Supplementary Table 4). These differences suggested the possibility of selection related to laboratory-data availability.

### 3.2 Cross-sectional association between CHG and CMM

Table 3 presents the logistic regression analysis of the association between CHG and CMM based on clinical data. In the clinical dataset analysis, each 1-SD increase in CHG was associated with greater odds of prevalent CMM across different models as follows: Model 1, 85% (OR = 1.85, 95% CI: 1.66–2.08); Model 2, 100% (OR = 2.00, 95% CI: 1.78–2.24); and Model 3, 96% (OR = 1.96, 95% CI: 1.74–2.21). In the fully adjusted Model 3, the odds of prevalent CMM were highest in Q4, whereas the estimates for Q2 and Q3 were not statistically significant. Using Q1 as the reference, the adjusted ORs (95% CIs) were 1.14 (0.96–1.36) for Q2, 1.18 (0.99–1.40) for Q3, and 2.34 (1.97–2.78) for Q4. Similarly, logistic regression analysis was conducted to examine the association between CHG and CMM using the CHARLS database (Table 4). In the CHARLS database analysis, each 1-SD increase in CHG was associated with greater odds of prevalent CMM across models: Model 1, 417% (OR = 5.17, 95% CI: 4.38–6.11); Model 2, 427% (OR = 5.27, 95% CI: 4.46–6.24); and Model 3, 314% (OR = 4.14, 95% CI: 3.46–4.94). In the fully adjusted Model 3, the odds of prevalent CMM were higher in Q3 and Q4, while the Q2 estimate was not statistically significant. With Q1 as the reference, the adjusted ORs (95% CIs) were 1.15 (0.73–1.81) for Q2, 1.79 (1.18–2.71) for Q3, and 6.07 (4.19–8.80) for Q4.

**Table 3:** Association between CHG and prevalent CMM in the clinical dataset.

CHG measure	Model 1		Model 2		Model 3	
	OR (95% CI)	*p* value	OR (95% CI)	*p* value	OR (95% CI)	*p* value
CHG (per 1 SD)	1.85 (1.66—2.08)	<0.001	2.00 (1.78—2.24)	<0.001	1.96 (1.74—2.21)	<0.001
CHG quartile						
1	1 (Ref)		1 (Ref)		1 (Ref)	
2	1.08 (0.91—1.27)	0.390	1.13 (0.95—1.34)	0.172	1.14 (0.96—1.36)	0.136
3	1.09 (0.92—1.29)	0.303	1.16 (0.98—1.38)	0.083	1.18 (0.99—1.40)	0.068
4	2.17 (1.85—2.55)	<0.001	2.37 (2.01—2.80)	<0.001	2.34 (1.97—2.78)	<0.001

Model 1 unadjusted; Model 2 adjusted for age, gender, educational level, smoking status, and drinking status; Model 3 adjusted for variables in Model 2 and BMI, SBP, WBC, CR, UA, hypertension, dyslipidemia and cancer.

OR, Odds Ratio; CI, Confidence Interval; Ref, reference.

**Table 4:** Association between CHG and prevalent CMM in CHARLS.

CHG measure	Model 1		Model 2		Model 3	
	OR (95% CI)	*p* value	OR (95% CI)	*p* value	OR (95% CI)	*p* value
CHG (per 1 SD)	5.17 (4.38—6.11)	<0.001	5.27 (4.46—6.24)	<0.001	4.14 (3.46—4.94)	<0.001
CHG quartile						
1	1 (Ref)		1 (Ref)		1 (Ref)	
2	1.26 (0.81—1.97)	0.309	1.25 (0.80—1.96)	0.330	1.15 (0.73—1.81)	0.541
3	2.11 (1.41—3.18)	<0.001	2.12 (1.41—3.19)	<0.001	1.79 (1.18—2.71)	0.006
4	8.78 (6.15—12.50)	<0.001	8.70 (6.09—12.40)	<0.001	6.07 (4.19—8.80)	<0.001

Model 1 unadjusted; Model 2 adjusted for age, gender, educational level, smoking status, and drinking status; Model 3 adjusted for variables in Model 2 and BMI, SBP, WBC, CR, UA, hypertension, dyslipidemia and cancer.

OR, Odds Ratio; CI, Confidence Interval; Ref, reference.

### 3.3 Nonlinear associations

RCS analyses showed nonlinear J-shaped associations between CHG and the odds of prevalent CMM in both datasets (*p* for non-linearity <0.001 for the unadjusted clinical model and both CHARLS models; *p* = 0.012 for the adjusted clinical model; Figure 2). In the clinical dataset (Figures 2A,B), two-piecewise logistic regression identified an estimated inflection point at CHG = 7.55 (Supplementary Table 5). Above this point, each unit increase in CHG was associated with higher odds of CMM (OR = 1.88, 95% CI: 1.09–3.23), whereas an inverse association was observed below the inflection point (OR = 0.22, 95% CI: 0.07–0.68). Bootstrap validation based on 1,000 resamples showed that the clinical inflection point was relatively stable, with a median estimate of 7.54 and a 95% bootstrap interval of 7.30–7.64 (Supplementary Table 6). In the CHARLS dataset (Figures 2C,D), the estimated inflection point was CHG = 6.13. Above this point, CHG was strongly associated with higher odds of CMM (OR = 8.11, 95% CI: 6.00–10.98), whereas the association below the inflection point was weaker and not statistically significant (OR = 1.49, 95% CI: 0.97–2.27). Bootstrap validation yielded a median inflection point of 6.12 (95% bootstrap interval: 4.72–6.16), indicating greater uncertainty in the exact location of the inflection point in CHARLS (Supplementary Table 6). Accordingly, these inflection points should be regarded as exploratory, dataset-specific estimates rather than established clinical thresholds.

**Figure 2:**
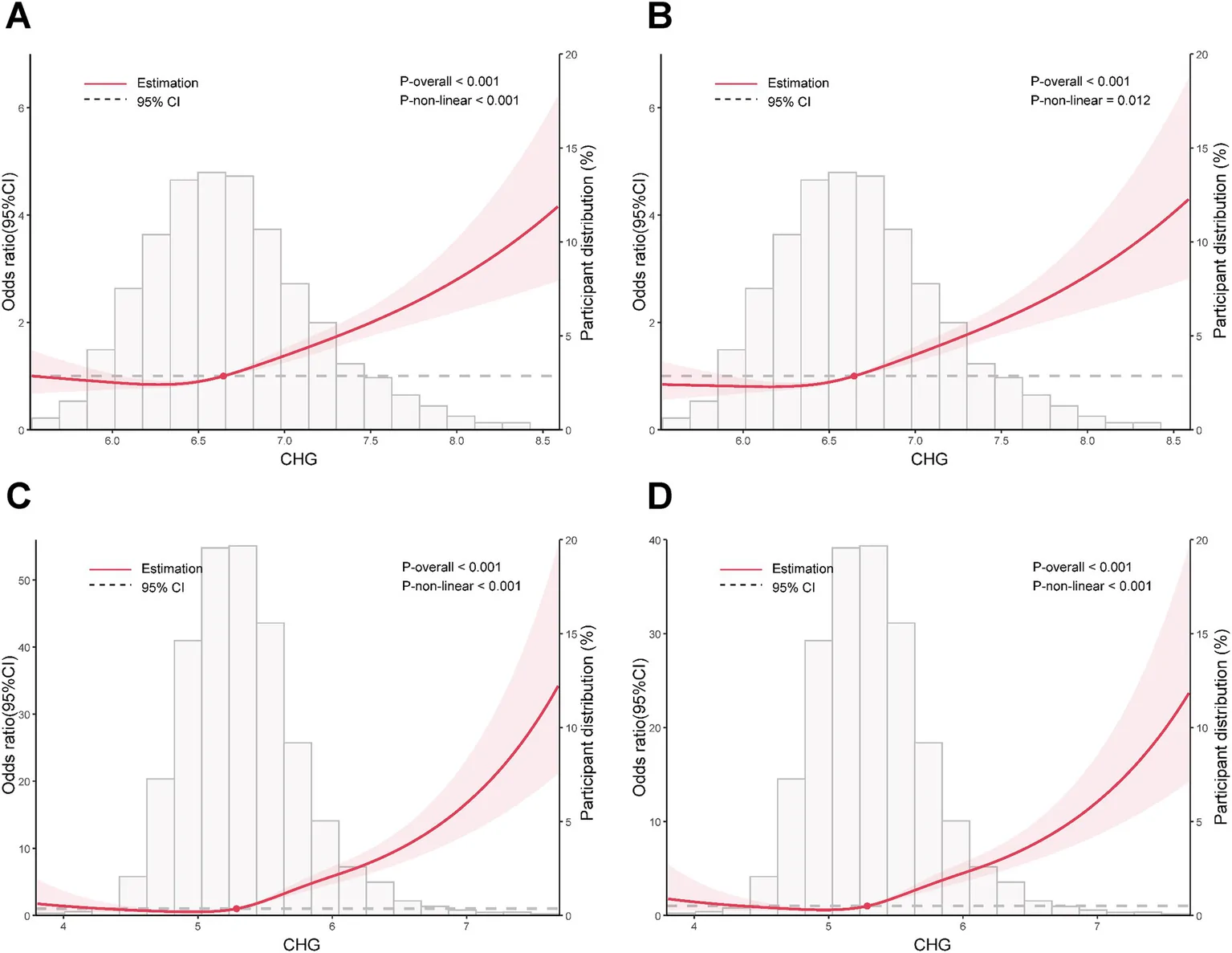
Restricted cubic spline associations between CHG and prevalent CMM. Panels **(A,B)** show the clinical dataset, and panels **(C,D)** show CHARLS. Panels **(A,C)** are unadjusted; panels **(B,D)** are adjusted for age, gender, education, smoking, drinking, BMI, SBP, WBC, CR, UA, hypertension, dyslipidemia, and cancer.

### 3.4 Discriminative performance analysis

Adding the CHG index to the basic covariate model increased CMM discrimination (Figure 3). In clinical data (Figure 3A), basic model AUC increased from 0.647 (95% CI: 0.631–0.662) to 0.690 (95% CI: 0.676–0.705, DeLong *p* < 0.001) upon adding CHG. This increment was greater than that obtained by adding TC (AUC = 0.651) or HDL-C (AUC = 0.651) (all *p* < 0.001), and was similar to that obtained by adding FBG (AUC = 0.697). In CHARLS data (Figure 3B), adding CHG raised AUC from 0.777 (95% CI: 0.755–0.799) to 0.834 (95% CI: 0.814–0.854, *p* < 0.001) (Supplementary Table 7). Single-index comparisons showed performance heterogeneity across populations (Supplementary Figure 2). In the CHARLS data (Supplementary Figure 2B), CHG had the highest standalone discrimination (AUC = 0.760, 95% CI: 0.735–0.786), exceeding TyG (0.733), METS-IR (0.725), AIP (0.672), NHHR (0.653), and RC (0.638). In clinical data (Supplementary Figure 2A), standalone CHG performance was lower (AUC = 0.585, 95% CI: 0.568–0.601), yielding discrimination comparable to TyG (AUC = 0.577, 95% CI: 0.561–0.594).

**Figure 3:**
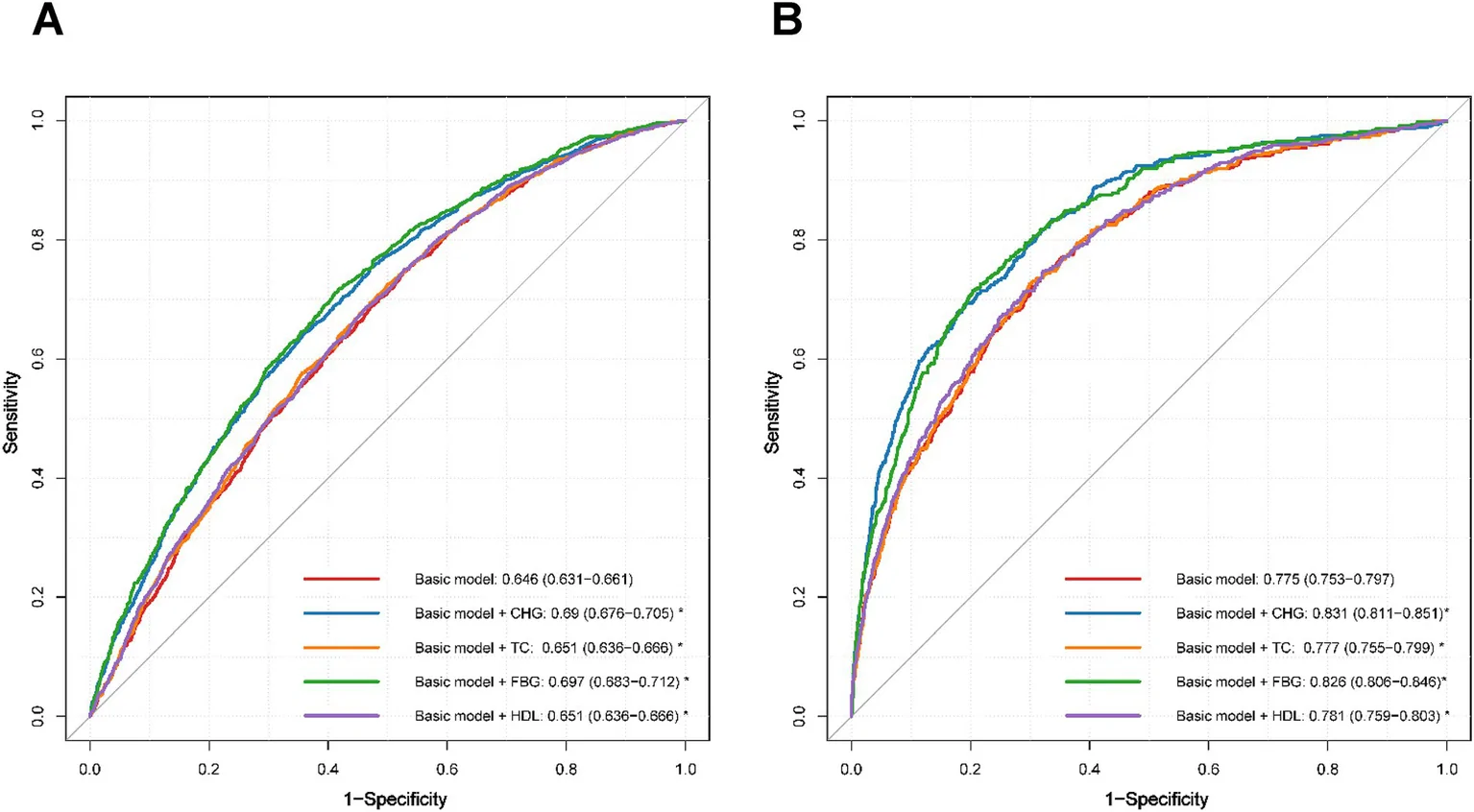
ROC curve analysis of CHG, TC, FBG, and HDL-C for discriminating prevalent CMM in the clinical dataset **(A)** and CHARLS **(B)**. The basic model was adjusted for age, gender, education, smoking, drinking, SBP, WBC, CR, UA, hypertension, dyslipidemia, and cancer. An asterisk indicates a statistically significant AUC difference from the basic model according to DeLong’s test (*p* < 0.05).

### 3.5 Subgroup analysis

The association between CHG and CMM was observed across the examined subgroups in both datasets. The smoking interaction in the clinical dataset was nominally significant (*p* for interaction = 0.043) but was not significant after false discovery rate correction (adjusted *p* = 0.344). No corrected interaction was significant in CHARLS (Figures 4, 5; Supplementary Table 8).

**Figure 4:**
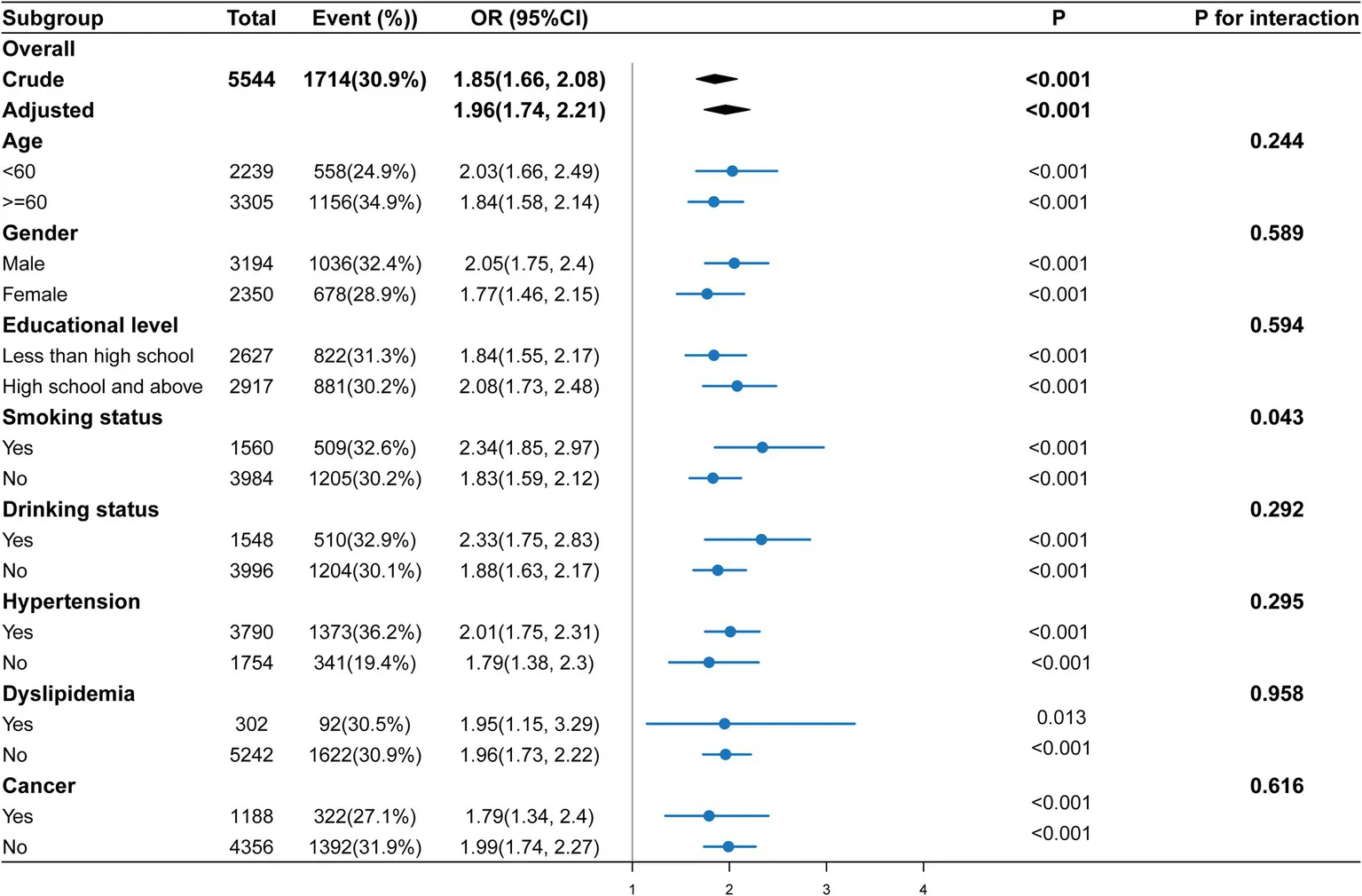
Subgroup analysis of the association between CHG and CMM in the clinical dataset.

**Figure 5:**
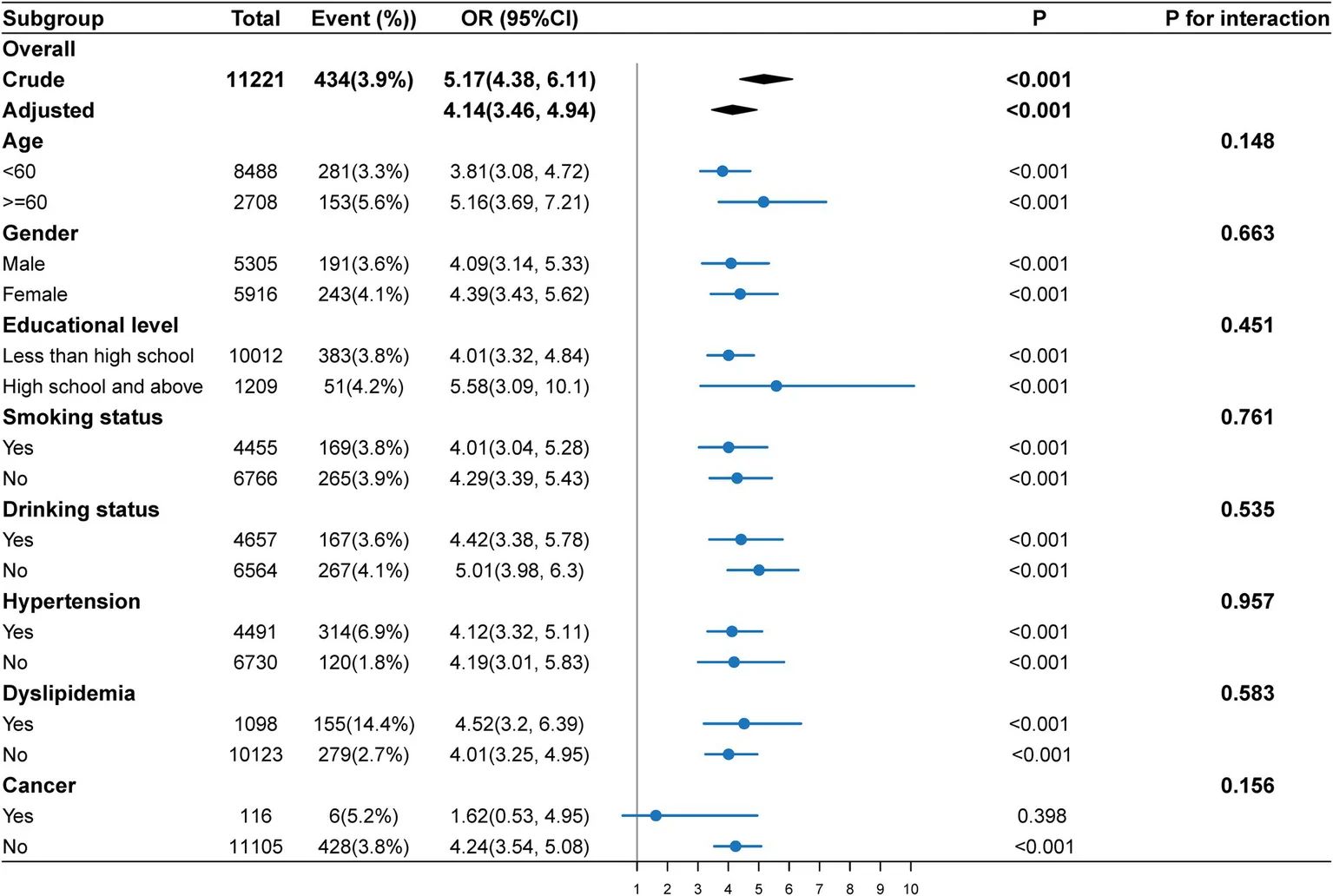
Subgroup analysis of the association between CHG and CMM in CHARLS.

### 3.6 Sensitivity analysis

Sensitivity analyses were generally consistent with the primary findings (Supplementary Tables 9, 10, 12). *E*-value sensitivity analysis showed point estimate *E*-values of 3.33 (lower limit: 2.87) for clinical data and 7.75 (lower limit: 6.38) for CHARLS data, suggesting that an unmeasured confounder would require associations of substantial magnitude with both CHG and CMM to explain the estimates (Supplementary Table 11). The only notable difference was observed in the phenotype-specific analysis in the clinical dataset, where heart disease plus stroke without diabetes showed an inverse association with CHG (OR = 0.75, 95% CI: 0.63–0.89; Supplementary Table 13), whereas the other phenotype-specific and sensitivity analyses remained consistent with the primary results.

## 4 Discussion

This study systematically evaluated the association between the CHG index and prevalent CMM using both clinical inpatient and CHARLS community datasets. Key findings include: (1) CHG is significantly and positively associated with CMM in both populations after multivariable adjustment; (2) a nonlinear J-shaped association was observed, with a higher inflection point in hospitalized patients (CHG = 7.55) than in community residents (CHG = 6.13); (3) adding CHG to the basic models increased discrimination.

Baseline analyses showed that across both datasets, participants with CMM were older, exhibited a higher prevalence of metabolic risk factors (such as hypertension and diabetes), and had higher CHG levels. Notably, cancer prevalence was lower among participants with CMM in the clinical dataset, whereas no corresponding difference was observed in CHARLS. This discrepancy may reflect inpatient selection or survival effects and should not be interpreted as evidence of a protective association. The CHG index integrates components associated with insulin resistance and metabolic dysregulation—including fasting blood glucose, total cholesterol, and HDL-C—which not only reflect metabolic health but are also associated with cardiovascular risk factors such as hypertension, obesity, and dyslipidemia (22). Multivariable logistic regression analyses showed that CHG was independently associated with prevalent CMM in both populations. Continuous CHG was significantly and positively associated with CMM across all adjusted models. Furthermore, quartile-stratified analyses showed that higher CHG quartiles were generally associated with greater odds of CMM, with the largest estimates observed in the highest quartile (Q4), supporting an overall increasing but non-linear association. Data from the National Health and Nutrition Examination Survey (2009–2018) showed that CHG is significantly and positively correlated with the prevalence of type 2 diabetes mellitus (30). Mo et al. (22) found that CHG independently predicts the incidence and prevalence of cardiovascular disease (CVD) among older adults in China. Zeng et al. (31) reported that elevated CHG levels are closely associated with stroke risk. Wei et al. (32) identified a significant U-shaped non-linear relationship between the CHG index and the incidence of metabolic syndrome (MetS). Therefore, consistent with a growing body of literature, the present findings are consistent with an association between CHG and CMM and suggest that CHG may provide complementary information for CMM assessment.

Insulin resistance is a hallmark feature of the metabolic syndrome, and is associated with metabolic abnormalities including hyperglycemia, dyslipidemia, and hypertension, which are also relevant to cardiovascular disease (33). The baseline characteristics of our study were consistent with this metabolic profile: in both the clinical and CHARLS populations, the CMM group exhibited elevated FBG, higher SBP, and dyslipidemia (Tables 1, 2), showing a pattern compatible with multidimensional metabolic dysregulation. These abnormalities are also relevant at the cellular and molecular levels. First, IR impairs endothelial and vascular smooth muscle function (34), reducing nitric oxide (NO) bioavailability and thereby promoting vasoconstriction (35), which aligns with the significantly higher SBP observed in the CMM group in this study. Notably, a marked reduction in HDL-C levels within the CMM group was consistently observed across both independent datasets, which was consistent with IR-related lipid dysmetabolism. Concurrently, IR is also associated with dyslipidemia characterized by elevated plasma triglycerides, increased LDL levels, and decreased HDL-C levels (36). Furthermore, IR has been linked to oxidative stress (37) and inflammatory pathway activation (38). In the present study, the significantly elevated WBC counts in the CMM group may reflect systemic inflammation. Collectively, these pathological processes may be relevant to the coexistence of cardiometabolic diseases.

Given the relevance of IR, identifying reliable surrogate biomarkers may support cardiometabolic assessment. The TyG index is the most commonly used surrogate marker for IR. Evidence from large-scale cohorts, including the UK Biobank, the China Health and Retirement Longitudinal Study (CHARLS), and the National Health and Nutrition Examination Survey (NHANES), consistently indicates that elevated or persistently high TyG index levels are significantly associated with an increased risk of CMM (39–41). However, the TyG index primarily reflects limited dimensions of glucolipid metabolism. In contrast, the CHG index, as a comprehensive indicator of IR, may provide complementary metabolic information due to its incorporation of total cholesterol and HDL levels. TC levels directly reflect circulating cholesterol burden and represent a well-established determinant of cardiovascular risk (42). Conversely, HDL-C levels are inversely correlated with cardiovascular risk (43). Low HDL-C levels not only signify reduced reverse cholesterol transport capacity, but also indicate compromised anti-inflammatory, antioxidant, antithrombotic, and endothelial protective functions (44). Dysfunctional HDL fails to effectively inhibit the adhesion and migration of T lymphocytes and monocytes toward the vascular endothelium and atherosclerotic sites (45, 46), thereby weakening anti-inflammatory control and cholesterol clearance capability. In the present study, the co-occurrence of reduced HDL-C levels and elevated WBC counts in the CMM group was consistent with concurrent lipid abnormalities and inflammation.

Such metabolic abnormalities have been associated with hepatic fat accumulation and adipose tissue inflammation. Inflammatory cytokines have also been linked to impaired insulin signaling and systemic insulin resistance, which are relevant to both diabetes and cardiovascular disease (47, 48). In the present study, the CHG index demonstrated a J-shaped relationship with CMM across both datasets (*p* for non-linearity <0.001 for the unadjusted clinical model and both CHARLS models; *p* = 0.012 for the adjusted clinical model). The estimated inflection points were 7.55 in the clinical dataset and 6.13 in CHARLS, above which the positive association with CMM became more pronounced. Bootstrap validation supported relatively good stability of the inflection point in the clinical dataset (median, 7.54; 95% bootstrap interval, 7.30–7.64), whereas greater uncertainty was observed in CHARLS (median, 6.12; 95% bootstrap interval, 4.72–6.16). The difference between datasets may partly reflect variation in metabolic profiles, disease burden, medication exposure, disease ascertainment, and the potential influence of acute illness on glucose and lipid measurements (49, 50). These inflection points should therefore be regarded as dataset-specific estimates rather than clinical cutoffs. The observed non-linear pattern may reflect the co-occurrence of insulin resistance, lipid metabolic dysfunction, and chronic inflammation as metabolic dysregulation becomes more pronounced.

ROC analyses showed that adding CHG to the basic model modestly but significantly improved discrimination, with the AUC increasing from 0.647 to 0.690 in the clinical dataset and from 0.777 to 0.834 in CHARLS. CHG also showed better discrimination than TC or HDL-C alone and performance comparable to FBG alone. However, the standalone discriminative ability of CHG differed substantially between the two populations, with a higher AUC in CHARLS than in the clinical dataset (0.760 vs. 0.585). This discrepancy may partly reflect differences between relatively stable community-dwelling participants and clinically heterogeneous hospitalized patients. Acute illness may induce transient stress hyperglycemia, while inpatient glucose levels can also be influenced by disease severity and active glycemic management (51, 52). Lipid measurements may likewise be modified by ongoing lipid-lowering treatment; randomized evidence has consistently shown that statins substantially alter circulating cholesterol concentrations, including reductions in total cholesterol and LDL-C and modest increases in HDL-C (53, 54). These factors could weaken the ability of a single CHG measurement to reflect habitual metabolic exposure in hospitalized patients. Consistent with this interpretation, additional adjustment for glucose-lowering and lipid-lowering medications attenuated the CHG–CMM association, although the association remained positive in both datasets. Thus, CHG may provide incremental discriminative information beyond conventional covariates, but its standalone performance appears to be population- and setting-dependent.

Although the sensitivity analyses were generally consistent with the primary findings, one notable exception was observed in the phenotype-specific analysis. Heart disease plus stroke without diabetes was inversely associated with CHG in the clinical dataset, whereas the corresponding association remained positive in CHARLS. Because fasting glucose is an integral component of CHG and previous studies have reported associations of CHG with glucose metabolic abnormalities and diabetes, the association of CHG may differ across CMM phenotypes according to the presence of diabetes (21). Moreover, acute illness and hospitalization can substantially alter glucose homeostasis and circulating lipid levels, potentially making a single inpatient CHG measurement less representative of habitual metabolic status (55). Lipid- and glucose-lowering treatment may further modify these components, which is consistent with the attenuation observed in our medication-adjusted analyses (53). Therefore, the discordant finding in this single phenotype may reflect differences in metabolic composition, treatment exposure, and inpatient case selection rather than a protective association of higher CHG. Given that the remaining sensitivity analyses were broadly consistent and no significant interactions remained after FDR correction, this isolated result should be interpreted cautiously as possible phenotype-specific heterogeneity.

Overall, these findings support a consistent association between the CHG index and CMM across both clinical and community populations. The difference in discriminative performance between the two datasets may reflect variation in population characteristics, comorbidity burden, treatment exposure, and metabolic stability. Although adding CHG modestly improved model discrimination, its standalone performance was substantially weaker in the clinical dataset, indicating that its discriminative contribution may be context-dependent. Subgroup analyses showed no significant interactions after FDR correction, while sensitivity analyses were largely consistent with the primary findings, with only limited heterogeneity observed in one phenotype-specific comparison. The non-linear associations observed in both datasets further suggest that the relationship between CHG and CMM may become stronger at higher CHG levels; however, the estimated inflection points should be interpreted as dataset-specific rather than clinically established thresholds. Taken together, CHG may serve as a simple and accessible marker of cardiometabolic burden, offering complementary information beyond established risk factors and may complement established approaches to CMM assessment.

From an aging and public health perspective, these findings may have implications for population health management. Because CHG is derived from routinely available laboratory measures, it may provide a low-burden adjunct for identifying middle-aged and older adults with a greater burden of clustered cardiometabolic conditions. In primary care and community health services, CHG could complement integrated assessment of glycemic status, lipid profile, blood pressure, comorbidity, medication use, and modifiable lifestyle factors. Such an approach is relevant to healthy aging, for which prevention and management of cardiometabolic multimorbidity require coordinated assessment across multiple conditions and care settings rather than a single-disease focus. Nevertheless, given the setting-dependent performance observed in this study, any CHG-informed application would require population-specific evaluation of calibration, clinical utility, feasibility, cost-effectiveness, and equity before broader implementation.

This study has several strengths. First, our study integrated two distinct data sources, a large-scale CHARLS dataset representative of the middle-aged and older population in China and real-world clinical data, allowing the consistency and setting-specific differences in the association to be examined. Second, our study employed a comprehensive analytical framework, including multivariable logistic regression with multivariable adjustment, quartile stratification, RCS curves for non-linear relationships, ROC curves, subgroup evaluations, and sensitivity analyses. This methodological approach enabled a detailed evaluation of the association between CHG and CMM. However, our study has several inherent limitations. First, the cross-sectional design precludes assessment of temporality and causal inference. Second, fasting glucose is both a component of CHG and part of diabetes ascertainment, which may strengthen associations for diabetes-containing outcomes through mathematical coupling. The phenotype-specific analyses reduce but do not eliminate this concern, and their results differed by setting. Third, disease ascertainment, laboratory methods, and treatment exposure were not harmonized between datasets. Fourth, missing CHG components may have introduced selection bias, and residual confounding remains possible. Finally, the observed discriminative performance should be interpreted within the context of the populations and settings included in this study, particularly given the differences between the clinical and community datasets. Longitudinal studies with more standardized measurements are warranted to further clarify the predictive value and generalizability of CHG for CMM.

## 5 Conclusion

The CHG index was independently associated with prevalent CMM in both community and inpatient Chinese populations and provided complementary discriminative information beyond conventional risk factors. As a simple marker derived from routinely available laboratory measures, CHG may help identify middle-aged and older adults with greater cardiometabolic burden and complement existing approaches to CMM assessment. These findings highlight its potential relevance to population health management and integrated chronic disease management in aging populations, while its setting-specific utility should be further evaluated before routine implementation.

## Data Availability

The CHARLS data used in this study are available from the China Health and Retirement Longitudinal Study database (http://charls.pku.edu.cn). The clinical data were collected by the First Affiliated Hospital of Xinjiang Medical University and are not publicly available because of patient privacy protections and restrictions imposed by the institutional ethics committee. Subject to approval by the relevant ethics committee, de-identified statistical data may be made available to academic researchers upon reasonable request to the corresponding authors.

## Glossary

CMM: cardiometabolic multimorbidity
CHG: cholesterol, high-density lipoprotein, and glucose index
TyG: triglyceride glucose index
CHARLS: China Health and Retirement Longitudinal Study
CMD: cardiometabolic disease
IR: insulin resistance
HIEG: hyperinsulinemic-euglycemic clamp
T2DM: type 2 diabetes mellitus
FBG: fasting blood glucose
TC: total cholesterol
HDL-C: high-density lipoprotein cholesterol
CVD: cardiovascular disease
SBP: systolic blood pressure
HbA1c: glycated hemoglobin
BMI: body mass index
WBC: white blood cell
LDL-C: low-density lipoprotein cholesterol
TG: triglyceride
CR: creatinine
UA: uric acid
SD: standard deviation
IQR: interquartile range
OR: Odds Ratio
CI: Confidence Interval
RCS: restricted cubic spline
ROC: receiver operating characteristic
AUC: area under the ROC curve
METS-IR: metabolic score for insulin resistance
AIP: atherogenic index of plasma
NHHR: non-HDL-C to HDL-C ratio
RC: Remnant Cholesterol
NHANES: National Health and Nutrition Examination Survey
UK Biobank: United Kingdom Biobank
MetS: metabolic syndrome
NO: nitric oxide
