# Supplementary material for: Independent association of the CHG index with cardiometabolic multimorbidity: real-world evidence from community and clinical datasets
Source: Front Public Health. 2026 Oct 7;14:1967131. doi: 10.3389/fpubh.2026.1967131 (PMC13683067; doi:10.3389/fpubh.2026.1967131)
Supplement: Supplementary file 1 [file Supplementary_file_1.DOCX]

Supplementary material

**Independent Association of the CHG Index with Cardiometabolic Multimorbidity: Real-World Evidence from Community and Clinical Datasets**

This file includes:

Supplementary tables

[Supplementary table 1 Distribution of missing data.	2](#_Toc240106467)

[Supplementary table 2 Collinearity Statistics.	3](#_Toc240106468)

[Supplementary table 3 Results of normality tests.	4](#_Toc240106469)

[Supplementary Table 4 Comparison of baseline characteristics between included participants and those excluded because of missing CHG components in the clinical and CHARLS data.	5](#_Toc240106470)

[Supplementary table 5 Comparison of Linear and Two-Piecewise Linear Regression Models for the Association between CHG and CMM.	7](#_Toc240106471)

[Supplementary Table 6 Bootstrap validation of the stability of CHG inflection points in the clinical and CHARLS data.	8](#_Toc240106472)

[Supplementary table 7 Pairwise comparison of ROC curves using DeLong's test.	9](#_Toc240106473)

[Supplementary Table 8 Subgroup interaction analyses with false discovery rate correction in the clinical and CHARLS data.	10](#_Toc240106474)

[Supplementary table 9 Complete-case sensitivity analysis of the association between the CHG index and CMM.	11](#_Toc240106475)

[Supplementary table 10 Sensitivity analysis of the association between the CHG index and CMM after excluding participants with chronic diseases at baseline.	12](#_Toc240106476)

[Supplementary table 11 E-value Sensitivity Analysis for Unmeasured Confounding.	13](#_Toc240106477)

[Supplementary Table 12 Sensitivity analysis of the association between the CHG index and CMM with additional adjustment for medication use in the clinical and CHARLS data.	14](#_Toc240106478)

[Supplementary Table 13 Association between the CHG index and specific cardiometabolic disease combinations in the clinical and CHARLS data.	15](#_Toc240106479)

Supplementary figure

[Supplementary figure 1	10](#_Toc235978320)

[Supplementary figure 2	11](#_Toc235978321)

Supplementary table 1 Distribution of missing data.

| **Database** | **Characteristics** | **No. of missing values** | **Percent (%)** |
| --- | --- | --- | --- |
| Clinical data | Age | 0 | 0 |
|  | Gender | 0 | 0 |
|  | Educational level | 0 | 0 |
|  | Smoking status | 0 | 0 |
|  | Drinking status | 0 | 0 |
|  | BMI | 111 | 2.0 |
|  | SBP | 4 | 0.1 |
|  | WBC | 162 | 2.9 |
|  | TG | 0 | 0 |
|  | LDL_C | 0 | 0 |
|  | CR | 17 | 0.31 |
|  | UA | 15 | 0.27 |
|  | Hypertension | 0 | 0 |
|  | Dyslipidemia | 0 | 0 |
|  | Cancer | 0 | 0 |
| CHARLS data | Age | 0 | 0 |
|  | Gender | 8 | 0.07 |
|  | Educational level | 10 | 0.09 |
|  | Smoking status | 5 | 0.04 |
|  | Drinking status | 6 | 0.05 |
|  | BMI | 1776 | 15.9 |
|  | SBP | 1697 | 15.2 |
|  | WBC | 274 | 2.44 |
|  | TG | 0 | 0 |
|  | LDL_C | 19 | 0.17 |
|  | CR | 4 | 0.04 |
|  | UA | 0 | 0 |
|  | Hypertension | 0 | 0 |
|  | Dyslipidemia | 209 | 1.86 |
|  | Cancer | 35 | 0.31 |

Supplementary table 2 Collinearity Statistics.

| **Database** | **Variable** | **GVIF** | **DF** | **GVIF^(1/(2*Df))** |
| --- | --- | --- | --- | --- |
| Clinical data | CHG | 1.619 | 1 | 1.272 |
|  | Age | 1.208 | 1 | 1.099 |
|  | Gender | 1.524 | 1 | 1.234 |
|  | Educational level | 1.134 | 1 | 1.065 |
|  | Smoking status | 3.197 | 1 | 1.788 |
|  | Drinking status | 3.177 | 1 | 1.782 |
|  | BMI | 1.031 | 1 | 1.015 |
|  | SBP | 1.202 | 1 | 1.096 |
|  | WBC | 1.053 | 1 | 1.026 |
|  | TG | 1.381 | 1 | 1.175 |
|  | LDL_C | 1.237 | 1 | 1.112 |
|  | CR | 1.092 | 1 | 1.045 |
|  | UA | 1.209 | 1 | 1.099 |
|  | Hypertension | 1.195 | 1 | 1.093 |
|  | Dyslipidemia | 1.086 | 1 | 1.042 |
|  | Cancer | 1.004 | 1 | 1.002 |
| CHARLS data | CHG | 2.002 | 1 | 1.415 |
|  | Age | 1.134 | 1 | 1.065 |
|  | Gender | 2.278 | 1 | 1.509 |
|  | Educational level | 1.074 | 1 | 1.036 |
|  | Smoking status | 1.823 | 1 | 1.35 |
|  | Drinking status | 1.405 | 1 | 1.185 |
|  | BMI | 1.004 | 1 | 1.002 |
|  | SBP | 1.516 | 1 | 1.231 |
|  | WBC | 1.025 | 1 | 1.013 |
|  | TG | 2.421 | 1 | 1.556 |
|  | LDL_C | 1.339 | 1 | 1.157 |
|  | CR | 1.223 | 1 | 1.106 |
|  | UA | 1.332 | 1 | 1.154 |
|  | Hypertension | 1.497 | 1 | 1.224 |
|  | Dyslipidemia | 1.036 | 1 | 1.018 |
|  | Cancer | 1.009 | 1 | 1.004 |

Supplementary table 3 Results of normality tests.

| **Database** | **Variable** | **Test** | **Statistic** | ***P -* value** |
| --- | --- | --- | --- | --- |
| Clinical data | Age | Lilliefors (Kolmogorov-Smirnov) | 0.388 | <0.001 |
|  | BMI | Lilliefors (Kolmogorov-Smirnov) | 0.373 | <0.001 |
|  | SBP | Lilliefors (Kolmogorov-Smirnov) | 0.387 | <0.001 |
|  | WBC | Lilliefors (Kolmogorov-Smirnov) | 0.294 | <0.001 |
|  | TC | Lilliefors (Kolmogorov-Smirnov) | 0.303 | <0.001 |
|  | TG | Lilliefors (Kolmogorov-Smirnov) | 0.228 | <0.001 |
|  | FBG | Lilliefors (Kolmogorov-Smirnov) | 0.303 | <0.001 |
|  | HDL_C | Lilliefors (Kolmogorov-Smirnov) | 0.307 | <0.001 |
|  | LDL_C | Lilliefors (Kolmogorov-Smirnov) | 0.257 | <0.001 |
|  | CR | Lilliefors (Kolmogorov-Smirnov) | 0.325 | <0.001 |
|  | UA | Lilliefors (Kolmogorov-Smirnov) | 0.296 | <0.001 |
|  | CHG | Lilliefors (Kolmogorov-Smirnov) | 0.434 | <0.001 |
| CHARLS data | Age | Lilliefors (Kolmogorov-Smirnov) | 0.406 | <0.001 |
|  | BMI | Lilliefors (Kolmogorov-Smirnov) | 0.364 | <0.001 |
|  | SBP | Lilliefors (Kolmogorov-Smirnov) | 0.381 | <0.001 |
|  | WBC | Lilliefors (Kolmogorov-Smirnov) | 0.304 | <0.001 |
|  | TC | Lilliefors (Kolmogorov-Smirnov) | 0.342 | <0.001 |
|  | TG | Lilliefors (Kolmogorov-Smirnov) | 0.229 | <0.001 |
|  | FBG | Lilliefors (Kolmogorov-Smirnov) | 0.353 | <0.001 |
|  | HDL_C | Lilliefors (Kolmogorov-Smirnov) | 0.292 | <0.001 |
|  | LDL_C | Lilliefors (Kolmogorov-Smirnov) | 0.278 | <0.001 |
|  | CR | Lilliefors (Kolmogorov-Smirnov) | 0.329 | <0.001 |
|  | UA | Lilliefors (Kolmogorov-Smirnov) | 0.301 | <0.001 |
|  | CHG | Lilliefors (Kolmogorov-Smirnov) | 0.425 | <0.001 |

Supplementary Table 4 Comparison of baseline characteristics between included participants and those excluded because of missing CHG components in the clinical and CHARLS data.

| **Dataset** | **Characteristic** | **Level** | **Excluded_missing_labs** | **Included** | **P value** | **SMD** | **Missing (%)** |
| --- | --- | --- | --- | --- | --- | --- | --- |
| **Clinical** | n |  | 870 | 5544 |  |  |  |
|  | Age, median [IQR] |  | 64.00 [55.00, 72.00] | 62.00 [55.00, 70.00] | 0.004 | 0.125 | 0.0 |
|  | Gender, n (%) | Female | 345 (39.5) | 2350 (42.3) | 0.127 | 0.058 | 0.0 |
|  |  | Male | 525 (60.5) | 3194 (57.7) |  |  |  |
|  | Education, n (%) | High school and above | 399 (45.8) | 2917 (52.6) | <0.001 | 0.137 | 0.0 |
|  |  | Less than high school | 471 (54.2) | 2627 (47.4) |  |  |  |
|  | Smoking, n (%) | No | 621 (71.7) | 3984 (71.5) | 0.680 | 0.017 | 0.0 |
|  |  | Yes | 249 (28.3) | 1560 (28.5) |  |  |  |
|  | Drinking, n (%) | No | 644 (74.4) | 3996 (72.1) | 0.291 | 0.041 | 0.0 |
|  |  | Yes | 226 (25.6) | 1548 (27.9) |  |  |  |
|  | BMI, median [IQR] |  | 25.53 [22.89, 28.31] | 25.86 [23.46, 28.41] | 0.076 | 0.043 | 2.8 |
|  | SBP, median [IQR] |  | 128.50 [120.00, 143.00] | 128.00 [119.00, 140.00] | 0.087 | 0.036 | 0.0 |
|  | WBC, median [IQR] |  | 6.52 [5.38, 8.14] | 6.24 [5.19, 7.49] | <0.001 | 0.163 | 4.2 |
|  | TG, median [IQR] |  | 120.46 [87.68, 170.05] | 129.31 [93.00, 187.77] | 0.003 | 0.069 | 5.6 |
|  | LDL, median [IQR] |  | 95.90 [74.25, 119.49] | 99.00 [75.41, 122.58] | 0.211 | 0.066 | 5.6 |
|  | CR, median [IQR] |  | 0.82 [0.66, 0.99] | 0.78 [0.66, 0.93] | 0.001 | 0.078 | 2.7 |
|  | UA, median [IQR] |  | 5.18 [4.24, 6.47] | 5.30 [4.40, 6.34] | 0.156 | 0.017 | 2.7 |
|  | Hypertension, n (%) | No | 290 (33.1) | 1754 (32.1) | 0.574 | 0.022 | 0.0 |
|  |  | Yes | 580 (66.9) | 3790 (68.4) |  |  |  |
|  | Dyslipidemia, n (%) | No | 823 (95.2) | 5242 (94.6) | 0.490 | 0.029 | 0.0 |
|  |  | Yes | 47 (4.8) | 302 (5.4) |  |  |  |
|  | Cancer, n (%) | No | 710 (82.1) | 4356 (78.6) | 0.012 | 0.096 | 0.0 |
|  |  | Yes | 160 (17.9) | 1188 (21.4) |  |  |  |
|  | Diabetes, n (%) | No | 627 (72.4) | 3461 (62.4) | <0.001 | 0.227 | 0.0 |
|  |  | Yes | 243 (27.6) | 2083 (37.6) |  |  |  |
|  | Heart disease, n (%) | No | 355 (40.7) | 2704 (48.8) | <0.001 | 0.181 | 0.0 |
|  |  | Yes | 515 (59.3) | 2840 (51.2) |  |  |  |
|  | Stroke, n (%) | No | 632 (73.0) | 4252 (76.7) | 0.005 | 0.103 | 0.0 |
|  |  | Yes | 238 (27.0) | 1292 (23.3) |  |  |  |
| **CHARLS** | n |  | 5700 | 11221 |  |  |  |
|  | Age, median [IQR] |  | 57.00 [50.00, 65.00] | 58.00 [52.00, 65.00] | 0.001 | 0.014 | 0.0 |
|  | Gender, n (%) | Female | 2742 (48.1) | 5910 (52.7) | <0.001 | 0.091 | 0.1 |
|  |  | Male | 2954 (51.8) | 5303 (47.3) |  |  |  |
|  |  | <NA> | 4 (0.1) | 8 (0.1) |  |  |  |
|  | Education, n (%) | High school and above | 952 (16.7) | 1208 (10.8) | <0.001 | 0.176 | 0.1 |
|  |  | Less than high school | 4736 (83.1) | 10003 (89.1) |  |  |  |
|  |  | <NA> | 12 (0.2) | 10 (0.1) |  |  |  |
|  | Smoking, n (%) | No | 3307 (58.0) | 6764 (60.3) | <0.001 | 0.073 | 0.1 |
|  |  | Yes | 2377 (41.7) | 4452 (39.7) |  |  |  |
|  |  | <NA> | 16 (0.3) | 5 (0.0) |  |  |  |
|  | Drinking, n (%) | No | 3288 (57.7) | 6562 (58.5) | <0.001 | 0.065 | 0.1 |
|  |  | Yes | 2393 (42.0) | 4653 (41.5) |  |  |  |
|  |  | <NA> | 19 (0.3) | 6 (0.1) |  |  |  |
|  | BMI, median [IQR] |  | 22.92 [20.63, 25.49] | 23.14 [20.82, 25.81] | 0.002 | 0.012 | 22.3 |
|  | SBP, median [IQR] |  | 127.67 [116.00, 143.67] | 127.67 [115.00, 143.00] | 0.330 | 0.012 | 21.5 |
|  | WBC, median [IQR] |  | 6.00 [4.88, 7.40] | 6.00 [4.99, 7.20] | 0.900 | 0.052 | 34.3 |
|  | TG, median [IQR] |  | 96.91 [78.32, 177.22] | 106.20 [75.22, 156.65] | 0.867 | 0.007 | 33.6 |
|  | LDL, median [IQR] |  | 103.22 [85.54, 121.20] | 114.05 [93.17, 137.24] | 0.088 | 0.308 | 33.6 |
|  | CR, median [IQR] |  | 0.79 [0.67, 1.11] | 0.76 [0.66, 0.88] | 0.503 | 0.345 | 33.7 |
|  | UA, median [IQR] |  | 4.49 [3.78, 5.14] | 4.33 [3.59, 5.19] | 0.335 | 0.170 | 33.5 |
|  | Hypertension, n (%) | No | 3710 (65.1) | 6730 (60.0) | <0.001 | 0.106 | 0.0 |
|  |  | Yes | 1990 (34.9) | 4491 (40.0) |  |  |  |
|  | Dyslipidemia, n (%) | No | 5138 (90.1) | 9937 (88.6) | 0.004 | 0.055 | 1.7 |
|  |  | Yes | 483 (8.5) | 1075 (9.6) |  |  |  |
|  |  | <NA> | 79 (1.4) | 209 (1.9) |  |  |  |
|  | Cancer, n (%) | No | 5635 (98.9) | 11070 (98.7) | 0.242 | 0.029 | 0.3 |
|  |  | Yes | 55 (1.0) | 116 (1.0) |  |  |  |
|  |  | <NA> | 10 (0.2) | 35 (0.3) |  |  |  |
|  | Diabetes, n (%) | No | 5378 (94.4) | 9263 (82.6) | <0.001 | 0.376 | 0.0 |
|  |  | Yes | 322 (5.6) | 1958 (17.4) |  |  |  |
|  | Heart disease, n (%) | No | 5050 (88.6) | 9818 (87.5) | 0.041 | 0.034 | 0.0 |
|  |  | Yes | 650 (11.4) | 1403 (12.5) |  |  |  |
|  | Stroke, n (%) | No | 5562 (97.6) | 10954 (97.6) | 0.909 | 0.003 | 0.0 |
|  |  | Yes | 138 (2.4) | 267 (2.4) |  |  |  |

CHG, cholesterol-high-density lipoprotein-glucose index; IQR, interquartile range; SMD, standardized mean difference; BMI, body mass index; SBP, systolic blood pressure; WBC, white blood cell count; TG, triglycerides; LDL, low-density lipoprotein cholesterol; CR, creatinine; UA, uric acid. Missing (%) indicates the proportion of missing observations for each characteristic.

Supplementary table 5 Comparison of Linear and Two-Piecewise Linear Regression Models for the Association between CHG and CMM.

| **Database** | **Model Type** | **Breakpoint** | **Segment** | **OR (95% CI)** | ***P* value** | ***P* for LRT** |  |
| --- | --- | --- | --- | --- | --- | --- | --- |
| Clinical data | One-line Linear | None | Entire range | 1.96 (1.74–2.21) | <0.001 | — |  |
|  | Two-piecewise | 7.55 | CHG > 7.55 | 1.88 (1.09–3.23) | 0.023 | <0.001 |  |
|  |  |  | CHG < 7.55 | 0.22 (0.07–0.68) | 0.009 |  |  |
| CHARLS data | One-line Linear | None | Entire range | 4.14 (3.46–4.94) | <0.001 | — |  |
|  | Two-piecewise | 6.13 | CHG > 6.13 | 8.11 (6.00–10.98) | <0.001 | <0.001 |  |
|  |  |  | CHG < 6.13 | 1.49 (0.97–2.27) | 0.068 |  |  |
| OR, Odds Ratio; CI, Confidence Interval; Ref, reference | | | | | | | |

Supplementary Table 6 Bootstrap validation of the stability of CHG inflection points in the clinical and CHARLS data.

| **Dataset** | **Original inflection point** | **Bootstrap median** | **95% bootstrap interval** | **Successful resamples** |
| --- | --- | --- | --- | --- |
| Clinical data | 7.55 | 7.54 | 7.30–7.64 | 1000/1000 |
| CHARLS data | 6.13 | 6.12 | 4.72–6.16 | 1000/1000 |

Inflection-point stability was assessed using 1,000 bootstrap resamples with replacement. The inflection point was re-estimated in each bootstrap sample using the same piecewise logistic regression procedure as in the primary analysis. The bootstrap median and the 2.5th–97.5th percentile interval of the bootstrap distribution are reported. Successful resamples indicate the number of bootstrap samples in which an inflection point was successfully estimated.

CHG, cholesterol–high-density lipoprotein–glucose index; CI, confidence interval.

Supplementary table 7 Pairwise comparison of ROC curves using DeLong's test.

| Clinical data | Models | Basic model | Basic + CHG | Basic + TC | Basic + FBG | Basic + HDL |
| --- | --- | --- | --- | --- | --- | --- |
|  | Basic model | — | < 0.001 | 0.0197 | < 0.001 | 0.0232 |
|  | Basic + CHG | < 0.001 | — | < 0.001 | 0.0176 | < 0.001 |
|  | Basic + TC | 0.0197 | < 0.001 | — | < 0.001 | 0.9547 |
|  | Basic + FBG | < 0.001 | 0.0176 | < 0.001 | — | < 0.001 |
|  | Basic + HDL | 0.0232 | < 0.001 | 0.9547 | < 0.001 | — |
| CHARLS data | Basic model | — | < 0.001 | 0.0042 | < 0.001 | 0.1408 |
|  | Basic + CHG | < 0.001 | — | < 0.001 | 0.3399 | < 0.001 |
|  | Basic + TC | 0.0042 | < 0.001 | — | < 0.001 | 0.2574 |
|  | Basic + FBG | < 0.001 | 0.3399 | < 0.001 | — | < 0.001 |
|  | Basic + HDL | 0.1408 | < 0.001 | 0.2574 | < 0.001 | — |
| CHG, target biomarker CHG; TC, total cholesterol; FBG, fasting blood glucose; HDL, high-density lipoprotein cholesterol; ROC, receiver operating characteristic.  All *P*-values were derived from two-tailed DeLong's tests comparing the area under the receiver operating characteristic curves (AUCs) between the respective models. | | | | | | |

Supplementary Table 8 Subgroup interaction analyses with false discovery rate correction in the clinical and CHARLS data.

| **Subgroup** | **Clinical P for interaction** | **Clinical FDR-adjusted P** | **CHARLS P for interaction** | **CHARLS FDR-adjusted P** |
| --- | --- | --- | --- | --- |
| Age | 0.244 | 0.590 | 0.148 | 0.624 |
| Gender | 0.589 | 0.704 | 0.663 | 0.870 |
| Education | 0.594 | 0.704 | 0.451 | 0.870 |
| Smoking | 0.043 | 0.344 | 0.761 | 0.870 |
| Drinking | 0.292 | 0.590 | 0.535 | 0.870 |
| Hypertension | 0.295 | 0.590 | 0.957 | 0.957 |
| Dyslipidemia | 0.958 | 0.958 | 0.583 | 0.870 |
| Cancer | 0.616 | 0.704 | 0.156 | 0.624 |

P values were adjusted separately within each dataset using the Benjamini-Hochberg false discovery rate (FDR) procedure. No subgroup interaction remained statistically significant after FDR correction.

Supplementary table 9 Complete-case sensitivity analysis of the association between the CHG index and CMM.

|  | **Model 1** | | **Model 2** | | **Model 3** | |
| --- | --- | --- | --- | --- | --- | --- |
|  | **OR (95% CI)** | **P value** | **OR (95% CI)** | **P value** | **OR (95% CI)** | **P value** |
| Clinical data | | | | | | |
| CHG (per 1 SD) | 1.81 (1.62 — 2.04) | <0.001 | 1.94 (1.72 — 2.19) | <0.001 | 2.87 (2.46 — 3.35) | <0.001 |
| CHG quartile |  |  |  |  |  |  |
| 1 | 1(Ref ) |  | 1(Ref ) |  | 1(Ref ) |  |
| 2 | 1.09 (0.92 — 1.30) | 0.305 | 1.14 (0.96 — 1.36) | 0.135 | 1.46 (1.21 — 1.76) | <0.001 |
| 3 | 1.08 (0.91 — 1.28) | 0.395 | 1.14 (0.96 — 1.36) | 0.142 | 1.65 (1.35 — 2.00) | <0.001 |
| 4 | 2.13 (1.81 — 2.52) | <0.001 | 2.33 (1.97 — 2.76) | <0.001 | 3.71 (3.02 — 4.58) | <0.001 |
| CHARLS data | | | | | | |
| CHG (per 1 SD) | 5.88 (4.84 — 7.16) | <0.001 | 5.99 (4.92 — 7.31) | <0.001 | 9.31 (6.98 — 12.4) | <0.001 |
| CHG quartile |  |  |  |  |  |  |
| 1 | 1(Ref ) |  | 1(Ref ) |  | 1(Ref ) |  |
| 2 | 1.31 (0.79 — 2.21) | 0.299 | 1.24 (0.74 — 2.09) | 0.421 | 1.56 (0.91 — 2.72) | 0.107 |
| 3 | 2.18 (1.38 — 3.54) | 0.001 | 2.09 (1.32 — 3.39) | 0.002 | 2.83 (1.72 — 4.79) | <0.001 |
| 4 | 9.42 (6.37 — 14.5) | <0.001 | 8.64 (5.83 — 13.4) | <0.001 | 12.5 (7.76 — 21.0) | <0.001 |
| Model 1 Unadjusted; Model 2 adjusted for age, gender, educational level, smoking status, and drinking status; Model 3 adjusted for variables in Model 2 and BMI, SBP, WBC, TG, LDL-C, CR, UA and hypertension  OR, Odds Ratio; CI, Confidence Interval; Ref, reference | | | | | | |

Supplementary table 10 Sensitivity analysis of the association between the CHG index and CMM after excluding participants with chronic diseases at baseline.

|  | **Model 1** | | **Model 2** | | **Model 3** | |
| --- | --- | --- | --- | --- | --- | --- |
|  | **OR** **(95% CI)** | **P value** | **OR** **(95% CI)** | **P value** | **OR** **(95% CI)** | **P value** |
| Clinical data | | | | | | |
| CHG (per 1 SD) | 1.63 (1.24 — 2.15) | <0.001 | 1.76 (1.32 — 2.34) | <0.001 | 1.81 (1.35 — 2.43) | <0.001 |
| CHG quartile |  |  |  |  |  |  |
| 1 | 1(Ref ) |  | 1(Ref ) |  | 1(Ref ) |  |
| 2 | 1.51 (1.02 — 2.25) | 0.043 | 1.58 (1.06 — 2.39) | 0.027 | 1.59 (1.06 — 2.41) | 0.027 |
| 3 | 1.55 (1.04 — 2.32) | 0.033 | 1.68 (1.12 — 2.55) | 0.013 | 1.68 (1.10 — 2.56) | 0.016 |
| 4 | 1.94 (1.30 — 2.92) | 0.001 | 2.14 (1.41 — 3.28) | <0.001 | 2.21 (1.44 — 3.41) | <0.001 |
| CHARLS data | | | | | | |
| CHG (per 1 SD) | 4.48 (3.13 — 6.36) | <0.001 | 4.52 (3.15 — 6.45) | <0.001 | 4.48 (3.08 — 6.48) | <0.001 |
| CHG quartile |  |  |  |  |  |  |
| 1 | 1(Ref ) |  | 1(Ref ) |  | 1(Ref ) |  |
| 2 | 1.19 (0.52 — 2.74) | 0.685 | 1.20 (0.52 — 2.76) | 0.670 | 1.19 (0.52 — 2.75) | 0.685 |
| 3 | 2.11 (1.02 — 4.61) | 0.049 | 2.17 (1.04 — 4.74) | 0.042 | 2.14 (1.03 — 4.70) | <0.047 |
| 4 | 7.01 (3.77 — 14.3) | <0.001 | 7.09 (3.80 — 14.5) | <0.001 | 6.84 (3.60 — 14.2) | <0.001 |
| Model 1 Unadjusted; Model 2 adjusted for age, gender, educational level, smoking status, and drinking status; Model 3 adjusted for variables in Model 2 and BMI, SBP, WBC, CR and UA  OR, Odds Ratio; CI, Confidence Interval; Ref, reference | | | | | | |

Supplementary table 11 E-value Sensitivity Analysis for Unmeasured Confounding.

| **Database** | **OR (95% CI)** | **E-value (Point Estimate)** | **E-value (Lower CI Limit)** |
| --- | --- | --- | --- |
| Clinical data | 1.96(1.74 – 2.21) | 3.33 | 2.87 |
| CHARLS data | 4.14(3.46 – 4.94) | 7.75 | 6.38 |

OR, Odds Ratio; CI, Confidence Interval; Ref, reference

Supplementary Table 12 Sensitivity analysis of the association between the CHG index and CMM with additional adjustment for medication use in the clinical and CHARLS data.

|  | **Model 1** | | | | **Model 2** | | | | **Model 3** | | | |
| --- | --- | --- | --- | --- | --- | --- | --- | --- | --- | --- | --- | --- |
|  | **OR (95% CI)** | | | **P value** | **OR (95% CI)** | | | **P value** | **OR (95% CI)** | | | **P value** |
| Clinical data |  | | |  |  | | |  |  | | |  |
| CHG (per 1 SD) | 1.85 (1.66–2.08) | | | <0.001 | 2.00 (1.78–2.24) | | | <0.001 | 1.65 (1.45–1.88) | | | <0.001 |
| CHG quartile |  | | |  |  | | |  |  | | |  |
| 1 | Ref | | | — | Ref | | | — | Ref | | | — |
| 2 | 1.08 (0.91–1.27) | | | 0.390 | 1.13 (0.95–1.34) | | | 0.172 | 1.09 (0.92–1.30) | | | 0.320 |
| 3 | 1.09 (0.92–1.29) | | | 0.303 | 1.16 (0.98–1.38) | | | 0.083 | 1.08 (0.90–1.29) | | | 0.399 |
| 4 | 2.17 (1.85–2.55) | | | <0.001 | 2.37 (2.01–2.80) | | | <0.001 | 1.86 (1.57–2.22) | | | <0.001 |
| CHARLS data | |  |  | | |  |  | | |  |  |  |
| CHG (per 1 SD) | 5.18 (4.39–6.12) | | | <0.001 | 5.28 (4.47–6.25) | | | <0.001 | 3.09 (2.56–3.72) | | | <0.001 |
| CHG quartile |  | | |  |  | | |  |  | | |  |
| 1 | Ref | | | — | Ref | | | — | Ref | | | — |
| 2 | 1.26 (0.81–1.98) | | | 0.308 | 1.25 (0.80–1.97) | | | 0.328 | 1.23 (0.78–1.94) | | | 0.378 |
| 3 | 2.12 (1.42–3.21) | | | <0.001 | 2.12 (1.42–3.23) | | | <0.001 | 1.86 (1.24–2.86) | | | 0.004 |
| 4 | 8.85 (6.29–12.8) | | | <0.001 | 8.76 (6.22–12.7) | | | <0.001 | 5.04 (3.51–7.45) | | | <0.001 |

Model 1 Unadjusted; Model 2 adjusted for age, gender, educational level, smoking status, and drinking status; Model 3 adjusted for variables in Model 2 and BMI, SBP, WBC, CR and UA

OR, Odds Ratio; CI, Confidence Interval; Ref, reference.

Supplementary Table 13 Association between the CHG index and specific cardiometabolic disease combinations in the clinical and CHARLS data.

| **Dataset** | **CMM phenotype** | **CHG category** | **Model 1 OR (95% CI)** | **P value** | **Model 2 OR (95% CI)** | **P value** | **Model 3 OR (95% CI)** | **P value** |
| --- | --- | --- | --- | --- | --- | --- | --- | --- |
| Clinical | Heart disease + Stroke | CHG (per 1-SD) | 0.70 (0.60–0.82) | <0.001 | 0.77 (0.65–0.90) | 0.002 | 0.75 (0.63–0.89) | 0.001 |
|  |  | Q1 | Ref | — | Ref | — | Ref | — |
|  |  | Q2 | 0.82 (0.67–1.01) | 0.066 | 0.88 (0.71–1.08) | 0.225 | 0.85 (0.69–1.05) | 0.131 |
|  |  | Q3 | 0.77 (0.63–0.95) | 0.015 | 0.85 (0.69–1.05) | 0.130 | 0.81 (0.65–1.01) | 0.056 |
|  |  | Q4 | 0.60 (0.48–0.75) | <0.001 | 0.66 (0.53–0.83) | <0.001 | 0.64 (0.51–0.81) | <0.001 |
| Clinical | Diabetes + Stroke | CHG (per 1-SD) | 2.60 (2.21–3.06) | <0.001 | 2.90 (2.45–3.42) | <0.001 | 2.44 (2.03–2.94) | <0.001 |
|  |  | Q1 | Ref | — | Ref | — | Ref | — |
|  |  | Q2 | 1.19 (0.87–1.64) | 0.266 | 1.27 (0.93–1.75) | 0.136 | 1.24 (0.90–1.70) | 0.190 |
|  |  | Q3 | 1.83 (1.38–2.46) | <0.001 | 2.01 (1.51–2.71) | <0.001 | 1.92 (1.43–2.60) | <0.001 |
|  |  | Q4 | 3.54 (2.72–4.66) | <0.001 | 4.05 (3.10–5.36) | <0.001 | 3.27 (2.46–4.38) | <0.001 |
| Clinical | Diabetes +  Heart disease | CHG (per 1-SD) | 2.79 (2.45–3.19) | <0.001 | 2.88 (2.52–3.30) | <0.001 | 2.27 (1.96–2.64) | <0.001 |
|  |  | Q1 | Ref | — | Ref | — | Ref | — |
|  |  | Q2 | 1.27 (1.01–1.60) | 0.041 | 1.29 (1.03–1.63) | 0.029 | 1.26 (1.00–1.60) | 0.056 |
|  |  | Q3 | 1.73 (1.39–2.16) | <0.001 | 1.78 (1.43–2.22) | <0.001 | 1.65 (1.31–2.08) | <0.001 |
|  |  | Q4 | 3.99 (3.26–4.91) | <0.001 | 4.13 (3.37–5.10) | <0.001 | 3.10 (2.49–3.86) | <0.001 |
| CHARLS | Heart disease + Stroke | CHG (per 1-SD) | 2.66 (1.78–3.81) | <0.001 | 2.76 (1.84–3.98) | <0.001 | 2.54 (1.66–3.74) | <0.001 |
|  |  | Q1 | Ref | — | Ref | — | Ref | — |
|  |  | Q2 | 1.00 (0.37–2.72) | >0.999 | 1.01 (0.37–2.76) | 0.976 | 1.03 (0.37–2.88) | 0.947 |
|  |  | Q3 | 1.50 (0.62–3.84) | 0.373 | 1.59 (0.65–4.06) | 0.314 | 1.51 (0.61–3.98) | 0.378 |
|  |  | Q4 | 4.68 (2.29–10.8) | <0.001 | 4.81 (2.35–11.2) | <0.001 | 4.31 (2.04–10.4) | <0.001 |
| CHARLS | Diabetes + Stroke | CHG (per 1-SD) | 4.21 (3.09–5.66) | <0.001 | 4.41 (3.22–5.96) | <0.001 | 4.19 (3.03–5.72) | <0.001 |
|  |  | Q1 | Ref | — | Ref | — | Ref | — |
|  |  | Q2 | 1.75 (0.53–6.70) | 0.371 | 1.76 (0.53–6.72) | 0.369 | 1.83 (0.54–7.31) | 0.346 |
|  |  | Q3 | 3.51 (1.26–12.4) | 0.027 | 3.63 (1.30–12.8) | 0.023 | 3.54 (1.23–13.1) | 0.031 |
|  |  | Q4 | 13.5 (5.52–44.6) | <0.001 | 13.6 (5.54–45.0) | <0.001 | 12.7 (5.02–44.4) | <0.001 |
| CHARLS | Diabetes +  Heart disease | CHG (per 1-SD) | 5.56 (4.65–6.66) | <0.001 | 5.62 (4.69–6.75) | <0.001 | 5.36 (4.46–6.46) | <0.001 |
|  |  | Q1 | Ref | — | Ref | — | Ref | — |
|  |  | Q2 | 1.44 (0.85–2.49) | 0.181 | 1.42 (0.83–2.45) | 0.200 | 1.40 (0.82–2.43) | 0.220 |
|  |  | Q3 | 2.24 (1.38–3.74) | 0.001 | 2.22 (1.37–3.70) | 0.002 | 2.11 (1.29–3.54) | 0.004 |
|  |  | Q4 | 10.8 (7.18–17.1) | <0.001 | 10.5 (6.99–16.7) | <0.001 | 9.71 (6.39–15.5) | <0.001 |

Model 1 Unadjusted; Model 2 adjusted for age, gender, educational level, smoking status, and drinking status; Model 3 adjusted for variables in Model 2 and BMI, SBP, WBC, CR and UA

OR, Odds Ratio; CI, Confidence Interval; Ref, reference

Supplementary figure 1

Difference in distribution of CHG in CMM and non-CMM groups. (A) In the clinical data. (B) In the CHARLS data.

Supplementary figure 2

ROC curve analysis of CHG, TyG, METS-IR, AIP, NHHR and RC for predicting CMM: comparison between two data sets. In clinical data(A) In CHARLS data(B). * indicates significantly improved predictive performance versus the basic model by DeLong's test (p < 0.05)
